# Wealth disparity and frailty among community-dwelling older adults in India

**DOI:** 10.1186/s12889-022-14434-9

**Published:** 2022-11-18

**Authors:** Priya Saravanakumar, Arun Balachandran, T. Muhammad, Drishti Drishti, Shobhit Srivastava

**Affiliations:** 1 Faculty of Health, School of Nursing and Midwifery, University of Technology Sydney, Building 10, Level 7, 235 Jones St, Ultimo, NSW 2007 Australia; 2 Department of Sociology, University of Maryland, College Park, MD 20742 USA; 3 International Institute for Population Sciences, Mumbai, 400088 India; 4 Department of Survey Research & Data Analytics, International Institute for Population Sciences, Mumbai, 400088 India

**Keywords:** Socioeconomic inequality, Frailty, Older adults, LASI

## Abstract

**Background:**

Due to the vast socioeconomic diversity among its residents, studying health inequality in India is of particular interest. This study aimed to investigate the wealth-based inequalities in physical frailty and to quantify the contributions of potential predictors of frailty to this inequality.

**Methods:**

Data were drawn from the first wave of the Longitudinal Ageing Study in India (LASI) conducted during 2017–18. Logistic regression analysis was used to examine the association between wealth status and frailty. We used the concentration index to measure the magnitude of wealth-related inequality in frailty. A decomposition analysis based on the logit model was used to assess the contribution of each predictor to the total inequality.

**Results:**

The prevalence of physical frailty was significantly higher among the older adults in the poor group than in the non-poor group [Difference (poor vs. non-poor): 6.4%; *p* < 0.001]. Regression results indicated that older adults in the poorest group were 23% more likely to be physically frail than those in the richest category [Adjusted odds ratio (AOR) = 1.23; 95% confidence interval (CI): 1.11, 1.38]. The overall concentration index of frailty was 0.058 among the older adults, indicating that frailty is more concentrated among older adults with poor wealth status. Body mass index, wealth index, educational status, and region were the major and significant contributors to the socioeconomic status (SES) related inequalities in frailty.

**Conclusions:**

Results suggest the need for formulating effective prevention and intervention strategies to decelerate the development of physical frailty among older adults in India, especially those with poor socioeconomic background.

**Supplementary Information:**

The online version contains supplementary material available at 10.1186/s12889-022-14434-9.

## Background

Frailty is an age-related geriatric syndrome characterized as the progressive loss of the physiological reserve and lack of response to the stressors [1] and is strongly associated with different adverse health outcomes such as decreased mobility, incident disability, hospitalization, falls, and increased risk of mortality [2–5]. Frailty is still an evolving concept, and there is a lack of consensus on the diagnostic criteria of frailty in epidemiological investigations and clinical practices. However, the most used frailty phenotype was defined by Fried and colleagues [1], which identifies frailty by the presence of three or more of five components (weakness, slowness, unintentional weight loss, poor endurance, and energy, and low physical activity). Frailty is not an inevitable consequence of old age rather an avoidable condition [6]. Globally, around 10% of the older adults are affected by frailty, and its prevalence increases with advanced age [7]. Therefore, the prevention or postponement of frailty is essential to maintain the functioning and health of the older populations.

Health inequalities between socioeconomic groups persist in developing, and developed countries [8]; frailty is no exception. Different studies have reported that frailty prevalence was higher among older persons with lower education and lower income [9–13]. Additionally, several longitudinal studies have also found low socioeconomic status (SES) as a potential risk factor of increased frailty level among older adults [10, 14–16]. Identification of variations in the prevalence of frailty across socioeconomic subgroups generates opportunities to prevent health-related inequalities. Multiple studies have investigated the health inequalities for different components of SES, including education [17, 18], income [19, 20], and occupation [21, 22]. Although it has been well-recognized that wealth has potential health implications, fewer studies have investigated the impact of wealth status on frailty [23, 24].

The proportion of the older population is growing worldwide because of the gain in life expectancy and low fertility rates, and this growth is more rapid in developing countries [25]. India is no exception; the number of Indian older adults aged 60 years and above is expected to grow from 104 million (8.6%) in 2011 to 319 million (19.1%) by 2050 [26]. With the increasing older population, the number of (pre-)frail older adults is expected to rise rapidly; thus, identifying the people at the risk of frailty is of utmost importance [27]. Most studies investigating socioeconomic inequalities in frailty are available from the developed countries [11, 14]. Although frailty has been studied in developing countries [28, 29], fewer studies have focused on the socioeconomic inequalities in frailty – specifically, wealth based-inequalities in frailty. A study based on the WHO’s Study on global AGEing and adult health (SAGE) data reported that education and income are the protective factors against frailty among Indian older adults [28]. Therefore, it can be expected that, in the Indian scenario, reducing the income- or wealth-based inequalities in frailty can help to tackle the burden of frailty and, consequently, to achieve meaningful health benefits.

A recent systematic review has recommended that reducing socioeconomic inequalities in frailty among older adults should be a policy concern [30]. Apart from determining the presence of socioeconomic inequalities, gaining insights into the factors which contribute mostly to the socioeconomic inequalities in frailty is necessary to identify more vulnerable sub-populations as the target groups and to suggest possible interventions directing towards reducing frailty and its adverse effects. Even though frailty-based studies are increasing, little attention has been paid to understand wealth-based (i.e., wealth-based) inequality in frailty over time. Due to the vast socioeconomic diversity among its residents, studying health inequality in India is of particular interest. Till now, no study in India in authors’ knowledge has investigated the socioeconomic inequalities associated with physical frailty in old age. Thus, the study aimed to investigate the wealth-based inequalities in physical frailty among older adults and to quantify the contributions of potential predictors of frailty to this inequality.

## Methods

### Data

This study utilizes data from India’s first nationally representative longitudinal Ageing survey (LASI-2017-18) which investigates into the health, economics and social determinants and consequences of population ageing in India [31]. The representative sample included 72,250 adults aged 45 and above and their spouses across all states and union territories of India except Sikkim. The LASI adopts a multistage stratified area probability cluster sampling design to select the eventual units of observation. Households with at least one member aged 45 and above were taken as the eventual unit of observation. This study provides scientific evidence on demographics, household economic status, chronic health conditions, symptom-based health condition, functional and mental health, biomarkers, health care utilization, work and employment etc. It enables the cross-state analyses and the cross-national analyses of ageing, health, economic status, and social behaviours and has been designed to evaluate the effect of changing policies and behavioural outcomes in India. Detailed information on the sampling frame is available on the LASI WAVE-1 Report [31]. The effective sample size for the present study was 11,842 and 18,709 older adults aged 60 years and above in the poor and non-poor category (total 30,551 older adults after excluding 913 individuals due to incomplete information on physical frailty components) [31]. All the steps/ methods were performed in accordance with the relevant guidelines and regulations, established by the declaration of Helsinki.

### Variable description

#### Outcome variable

##### Frailty

The physical frailty among older adults were assessed using an adapted version of the frailty phenotype described by Fried and colleagues [32]. Multiple studies on Indian older adults utilized the Fried’s phenotype framework to define frailty [33–35]. The frailty phenotype developed by Fried and colleagues consists of five components: (1) self-reported exhaustion, (2) unintentional weight loss, (3) weak grip strength, (4) self-reported low physical activity, and (5) slow walking time. The overall score of frailty phenotype lies between 0 and 5. Respondents with a score of 0 to 2 were considered “non-frail,” and three or higher as “frail”. The assessment of the individual domains is described in Table 1. The criticism of the Fried’s criteria of defining frailty is that it focuses mainly on physical health deficits. Also, the Fried’s criteria for defining frailty do not account for the role of various psychological factors, including cognition and depression among older adults [36, 37]. In addition, the reliance of Fried’s criteria on questionnaires to identify weight loss and exhaustion potentially suffers from participant bias [38].

**Table 1:** Criteria used to define physical frailty in LASI, 2017–18

**Exhaustion**	During the past week, how often did you feel: (a) tired or low in energy; (b) that everything you did was an effort. Less than three days = 0; More than three days =1.	
**Grip strength**	Average grip strength score in dominant hand (2 trials) using Smedley’s handheld dynamometer (adjusted for gender and body mass index (BMI))	
	**BMI**	**Cut-off for Grip strength**
	**For men**	
	BMI ≤ 24	≤ 29 kg
	BMI 24.1–26	≤ 30 kg
	BMI 26.1–28	≤ 30 kg
	BMI > 28	≤ 32 kg
	**For women**	
	BMI ≤ 23	≤ 17 kg
	BMI 23.1–26	≤ 17.3 kg
	BMI 26.1–29	≤ 18 kg
	BMI > 29	≤ 21 kg
**Walk time**	In the LASI, respondents were asked to walk 4 m twice. The time taken to walk was recorded in seconds each time, and the average time taken in both trials was calculated. (Stratified by gender and height (gender-specific cut-off a medium height)	
		Cut-off for Time to Walk 4-m
	**Men**	
	Height ≤ 173 cm	≥ 7 seconds
	Height > 173 cm	≥ 6 seconds
	**Women**	
	Height ≤ 159 cm	≥ 7 seconds
	Height > 159 cm	≥ 6 seconds
**Weight loss**	Do you think that you have lost weight in the last 12 months because there was not enough food at your household?” Yes = 1, No = 0.	
**Physical activity**	How often do you take part in sports or vigorous activities, such as running or jogging, swimming, going to a health center or gym, cycling, or digging with a spade or shovel, heavy lifting, chopping, farm work, fast bicycling, cycling with loads? One to three times a month or hardly ever or never = 1, once a week or more than once a week = 0	
**Total score**	5 Items: 0–2 deficits = non-frail, 3+ deficits = frail Allowed missing 1–2 items, missing imputed with 0 (sum of available items)	

### Explanatory variable

#### Main explanatory variable

Wealth index was calculated using variables related to household assets, amenities, and housing quality. For constructing wealth index in the LASI, we have utilized a similar approach that is being used in Demographic Health Surveys (DHS) [39]. To construct wealth index, we utilized a set of 46 variables that covers the broad domains of the household’s wealth and amenities and access to financial institutions. Further details are provided in Supplementary file. For the analysis purposes, the wealth quintile was categorized as *poor* which includes poorest and poorer category and *non-poor* which includes middle, richer and richest.

#### Other explanatory variables

A set of potentially related covariates including individual, behavioural, health, and household factors were included in the analysis. Individual factors were age (young-old (60–69 years), old-old (70–79 years) and oldest-old (80+ years)); sex (male, female); educational status (no education/primary not completed, primary, secondary and higher); living arrangement (living alone, living with spouse only, living with children only and living with others); marital status (currently married, widowed and others (separated/divorced/never married)); working status (never worked, currently working, currently not working and retired); and social engagement (no, yes). The participants were categorized as socially engaged if they participated in any of these activities: eating out in a restaurant or hotel, going to park/beach, playing cards or indoor games, playing outdoor games, visiting relatives/friends, attending cultural performances, attending religious functions, attend political/community/organization group meetings, read books/newspapers, watch television, use a computer for e-mail/net surfing etc. Behavioral factors include tobacco consumption (no, yes) and alcohol consumption (no, yes). Health-related factors comprises of body mass index (< 18.5 kg/m^2^ (underweight), 18.5 to 24.9 kg/m^2^ (normal), ≥25 kg/m^2^ (overweight/obese) [40]); self-rated health (good, poor); difficulties in basic and instrumental activities of daily living (ADL and IADL) (no, yes); and morbidity status (0, 1, 2 and above). The morbid conditions that are considered to create the morbidity status are hypertension, coronary heart diseases, stroke, cancer, diabetes mellitus, chronic bone/joint diseases, neurological or psychiatric conditions, chronic lung diseases and high cholesterol. Basic ADLs included six activities: dressing, bathing, walking, eating, getting in or out of bed, and toileting, which were necessary for older individuals’ independent living in the community. On the other hand, IADLs included seven activities: preparing food, shopping, making telephone calls, taking medications, working around the house, managing money, and finding an address in an unfamiliar place [41]. Various household factors were: Religion (Hindu, Muslim, Christian, and Others), Caste (Scheduled Tribe, Scheduled Caste, Other Backward Class, and others), residence (rural and urban), and region (North, Central, East, Northeast, West, and South.

### Statistical analysis

Bivariate analysis was performed to identify the significant variables that are related to frailty. A two-sample proportion test was used to evaluate if the prevalence of the various socioeconomic and demographic variables obtained according to the wealth status (Poor, Non-poor) were significantly different. Multiple logistic regression was used to examine the association between frailty and various explanatory covariates. Logistic regression is used when the outcome variable is binary (no/yes (0/1)). The independent variables may be continuous or categorical or a mixture of the two. It is advantageous over simple linear regression in terms of the interpretation and indicates the relative likelihood of the event of interest. Our dependent variable, physical frailty status, has two categories: 0 = not frail; 1 = frail. Therefore, the utilization of logistic regression model is considered appropriate (See supplementary material for detailed mathematical expressions).

The presence of multicollinearity among the independent variables was detected using the Variance Inflation Factor (VIF) at a cut-off point of 10. In the final model, to check the goodness-of-fit, an F-adjusted goodness-of-fit test was employed. Due to complex sampling design effects in LASI, we accounted for inverse probability weights by using the *svyset* command in Stata 15.1. Model-1 represents the unadjusted estimates whereas Model-2 represents the adjusted estimates i.e., model-2 was adjusted for age, sex, education, living arrangement, working status, marital status, social engagement, tobacco consumption, alcohol consumption, BMI, self-rated health, ADL, IADL, morbidity status, religion, caste, place of residence and region.

Concentration curve (CC) and concentration index (CCI) were used to determine the inequalities in the distribution of the frailty by wealth index scores. The CC depicts how a cumulative share of the frailty (y-axis) is accounted for by the cumulative percentage of the individuals ranked by wealth scores (x-axis). If every individual has an identical health outcome, regardless of the wealth status, the CC would be a 45^0^ line that runs from the lower-left corner to the upper right corner, also known as the “line of equality.” On the contrary, if the health outcome variable has higher values among poorer people, the CC will lie above the “line of equality” and vice versa. The farther the curve is away from the baseline, represented by the equality line, the more unequal is the distribution of the health outcome variable [42]. The CCI corresponds to twice the area between the CC and the line of equality [43]. In the present paper, the CCI is computed as twice the covariance of the health outcome variable and a person’s rank in terms of wealth status, divided by the mean of the health variable [44]:

1 $$\textrm{CI}=\frac{2}{\upmu}\operatorname{cov}\left({\upgamma}_{\textrm{j}},{\textrm{R}}_{\textrm{j}}\right)$$

Where γ_j_ and R_j_ are the health status and fractional rank (in terms of the index of economic status) of the jth individual, respectively; μ is the mean of the health outcome variable, and cov denotes the covariance.

### Decomposition of the CCI

The present study used Wagstaff’s CCI decomposition approach to reveal the contribution of each explanatory variable to the measured health inequality (i.e., frailty inequality) [45]. According to Wagstaff, a linear regression model linking health outcome variable (y) to a set of k explanatory variables (x_k_):

2 $${\textrm{y}}_{\textrm{i}}=\upalpha +{\sum}_{\textrm{k}}{\upbeta}_{\textrm{k}}{\textrm{x}}_{\textrm{ki}}+{\upvarepsilon}_{\textrm{i}}$$

Where x_ki_ is a set of k explanatory variables for the ith individual, β_k_ signifies the coefficient, and ε_i_ is an error term. Given the association of y_i_ and x_ki_, in eq. (2), the concentration index (CCI) for y, can be written as follows:

3 $$\textrm{C}={\sum}_{\textrm{k}}\left(\frac{\upbeta_{\textrm{k}}{\overline{\textrm{x}}}_{\textrm{k}}}{\upmu}\right){\textrm{C}}_{\textrm{k}}+\frac{\textrm{GC}_{\upvarepsilon}}{\upmu}$$

Where C denotes the overall concentration index, μ is the mean of y, ${\overline{\textrm{x}}}_{\textrm{k}}$ is the mean of x_k_, C_k_ is the normalized concentration index for x_k_ (defined exactly like CCI), $\frac{\upbeta_{\textrm{k}}{\overline{\textrm{x}}}_{\textrm{k}}}{\upmu}$ is the elasticity of health variable with the explanatory variables, and GC_ε_ is the generalized CCI for *ε*_*i*_ (residual component). Equation (3) suggests that the concentration index consists of explained and residual (unexplained) components. In most cases, health outcome variables are rarely continuous. We have approximated decomposition analysis by using marginal effects on the logit model. A linear approximation of the non-linear estimation can be represented as:

4 $${\textrm{y}}_{\textrm{i}}={\upalpha}^{\textrm{m}}+{\sum}_{\textrm{k}}{\upbeta}_{\textrm{k}}^{\textrm{m}}{\textrm{x}}_{\textrm{ki}}+{\upmu}_{\textrm{i}}$$

Where ${\upbeta}_{\textrm{k}}^{\textrm{m}}$ is the marginal effects ($\frac{\textrm{dy}}{\textrm{dx}}$) of each x; μ_i_ signifies the error term generated by the linear approximation. The concentration index for the heath variable (y) (in our case, frailty) is given as:

5 $$\textrm{CI}={\sum}_{\textrm{k}}\left(\frac{\upbeta_{\textrm{k}}{\overline{\textrm{x}}}_{\textrm{k}}}{\upmu}\right){\textrm{C}}_{\textrm{k}}+\textrm{GC}_{\upvarepsilon}/\upmu$$

## Results

In total, 30,551 participants comprise the effective sample, including 11,842 older adults in the poor group and 18,709 in the non-poor group. Table 2 shows the socio-economic and demographic profiles of the two groups. The pattern of age and sex distribution was similar among both poor and non-poor groups. The proportion of illiterate (84.3% vs. 54.3%), living alone (9.8% vs. 2.3%), non-working (39.4% vs. 32.7%), and widowed (38.2% vs. 34.2%) older adults in the poor group was higher than that in the non-poor group. The level of social engagement was higher among older adults in the non-poor group than in the poor group (94.3% vs. 88.0%). The proportion of tobacco (47.1% vs. 34.4%) and alcohol (16.6% vs. 13.0%) consumption were higher among older adults in the poor group than the non-poor group. According to different health-related predictors, the non-poor group had a higher proportion of older adults who were overweight/obese (28.4% vs. 10.7%), had good self-rated health (55.7% vs. 46.4%), and had no difficulty in ADL (79.2% vs. 74.8%) and IADL (56.8% vs. 47.3%) than in the non-poor group. Moreover, the proportion of older adults with more than two comorbidities was higher in the non-poor group than in the poor group (29.8% vs. 16.7%).

**Table 2:** Socio-economic and demographic profile of the study population in India, 2017–18

Background characteristics	Poor		Non-poor	
	Sample	Percentage	Sample	Percentage
**Individual factors**				
**Age**				
Young-old (60–69 years)	7034	59.4	11,125	59.5
Old-old (70–79 years)	3525	29.8	5602	29.9
Oldest-old (80+ years)	1283	10.8	1982	10.6
**Sex**				
Male	5413	45.7	9076	48.5
Female	6429	54.3	9633	51.5
**Education**				
No education/primary not completed	9987	84.3	10,149	54.3
Primary completed	914	7.7	2653	14.2
Secondary completed	804	6.8	3654	19.5
Higher and above	137	1.2	2253	12.0
**Living arrangements**				
Alone	1156	9.8	423	2.3
With spouse only	2973	25.1	2844	15.2
With children only	6972	58.9	14,579	77.9
Others	741	6.3	862	4.6
**Marital status**				
Currently married	7039	59.4	11,956	63.9
Widowed	4523	38.2	6394	34.2
Others	280	2.4	360	1.9
**Working status**				
Never worked	2489	21.0	5799	31.0
Currently working	4429	37.4	4599	24.6
Currently not working	4667	39.4	6112	32.7
Retired	256	2.2	2198	11.7
**Social engagement**				
No	1417	12.0	1072	5.7
Yes	10,425	88.0	17,637	94.3
**Behavioral factors**				
**Tobacco consumption**				
No	6261	52.9	12,269	65.6
Yes	5581	47.1	6440	34.4
**Alcohol consumption**				
No	9882	83.5	16,273	87.0
Yes	1960	16.6	2436	13.0
**Health factors**				
**Body mass index**^a^				
Underweight	4200	35.5	2785	14.9
Normal	5393	45.5	8919	47.7
Overweight/obese	1262	10.7	5313	28.4
**Self-rated health**				
Good	5498	46.4	10,424	55.7
Poor	6344	53.6	8285	44.3
**Difficulty in ADL**				
No	8853	74.8	14,809	79.2
Yes	2989	25.2	3900	20.9
**Difficulty in IADL**				
No	5599	47.3	10,616	56.8
Yes	6243	52.7	8093	43.3
**Morbidity status**				
0	6711	56.7	7241	38.7
1	3157	26.7	5885	31.5
2+	1974	16.7	5583	29.8
**Household factors**				
**Religion**				
Hindu	9899	83.6	15,328	81.9
Muslim	1292	10.9	1978	10.6
Christian	343	2.9	542	2.9
Others	308	2.6	861	4.6
**Caste**				
Scheduled Caste	2957	25.0	2600	13.9
Scheduled Tribe	1469	12.4	813	4.4
Other Backward Class	5175	43.7	8716	46.6
Others	2241	18.9	6580	35.2
**Place of residence**				
Rural	10,566	89.2	10,370	55.4
Urban	1276	10.8	8339	44.6
**Region**				
North	896	7.6	3224	17.2
Central	3143	26.5	3020	16.1
East	3814	32.2	3109	16.6
Northeast	356	3.0	554	3.0
West	1597	13.5	3811	20.4
South	2037	17.2	4991	26.7
**Total**	11,842	100	18,709	100

^a^sample was low due to missing cases

Table 3 shows the bivariate analysis of percentages distribution of frailty among older adults in poor and non-poor groups. The prevalence of frailty was significantly higher among the older adults in the poor group than in the non-poor group [Difference (poor vs. non-poor): 6.4%; *p* < 0.001]. Irrespective of age, sex, education, living arrangements, working status, marital status, and social engagements, the poor group has a significantly higher prevalence of frail older adults than the non-poor group. For both poor and non-poor groups, the prevalence of frailty was higher in the older adults who were underweight, never consumed tobacco or alcohol, had poor self-rated health, had difficulties in ADL and IADL, and had more than two comorbidities.

**Table 3:** Percentage of frail older adults by their background characteristics in India, 2017–18

Background characteristics	Poor	Non-poor	Difference	***p***-value
	%	%	%	
**Individual factors**				
**Age**				
Young-old (60–69 years)	24.6	18.8	5.8	0.001
Old-old (70–79 years)	41.8	34.9	6.9	0.001
Oldest-old (80+ years)	58.2	50.4	7.8	0.537
**Sex**				
Male	31.3	24.2	7.0	0.001
Female	35.1	29.5	5.6	0.001
**Education**				
No education/primary not completed	34.7	31.0	3.7	0.051
Primary completed	30.8	28.3	2.5	0.210
Secondary completed	22.2	19.7	2.5	0.002
Higher and above	18.9	19.0	−0.1	0.894
**Living arrangements**				
Alone	42.7	32.7	10.0	0.112
With spouse only	29.4	24.3	5.1	0.001
With children only	32.6	26.5	6.0	0.001
Others	41.9	39.5	2.4	0.878
**Marital status**				
Currently married	28.7	22.6	6.1	0.001
Widowed	40.7	35.3	5.3	0.002
Others	33.4	23.3	10.1	0.309
**Working status**				
Never worked	36.5	30.4	6.2	0.001
Currently working	16.0	13.0	3.0	0.005
Currently not working	47.6	36.2	11.4	0.001
Retired	42.6	21.5	21.1	0.001
**Social engagement**				
No	42.7	39.6	3.1	0.023
Yes	32.1	26.2	5.9	0.004
**Behavioral factors**				
**Tobacco consumption**				
No	34.0	27.5	6.5	0.001
Yes	32.6	25.9	6.7	0.001
**Alcohol consumption**				
No	34.3	27.5	6.8	0.001
Yes	28.8	23.5	5.2	0.001
**Health factors**				
**Body mass index**				
Underweight	42.8	38.4	4.3	0.363
Normal	33.0	28.7	4.3	0.002
Overweight/obese	24.5	26.2	−1.7	0.033
**Self-rated health**				
Good	25.1	20.6	4.5	0.001
Poor	40.5	34.9	5.6	0.001
**Difficulty in ADL**				
No	28.1	23.4	4.7	0.001
Yes	48.9	40.4	8.5	0.001
**Difficulty in IADL**				
No	24.2	20.5	3.6	0.001
Yes	41.6	35.4	6.2	0.001
**Morbidity status**				
0	29.7	23.3	6.4	0.001
1	35.8	26.9	8.9	0.001
2+	41.8	31.7	10.1	0.001
**Household factors**				
**Religion**				
Hindu	32.9	26.8	6.1	0.001
Muslim	36.9	30.8	6.1	0.001
Christian	33.3	25.8	7.5	0.073
Others	33.8	22.3	11.5	0.002
**Caste**				
Scheduled Caste	34.6	27.1	7.5	0.001
Scheduled Tribe	27.9	25.4	2.6	0.017
Other Backward Class	35.2	26.8	8.4	0.001
Others	31.0	27.3	3.7	0.001
**Place of residence**				
Rural	33.7	27.3	6.4	0.001
Urban	30.7	26.6	4.2	0.001
**Region**				
North	27.3	26.7	0.6	0.380
Central	34.5	29.1	5.4	0.015
East	35.1	32.5	2.6	0.843
Northeast	26.6	19.9	6.6	0.012
West	27.2	20.9	6.3	0.009
South	37.0	27.8	9.1	0.001
**Total**	**33.4**	**27.0**	**6.4**	**0.001**

Differences: Poor - Non poor; *p*-value based on proportion test

**Fig. 1:**
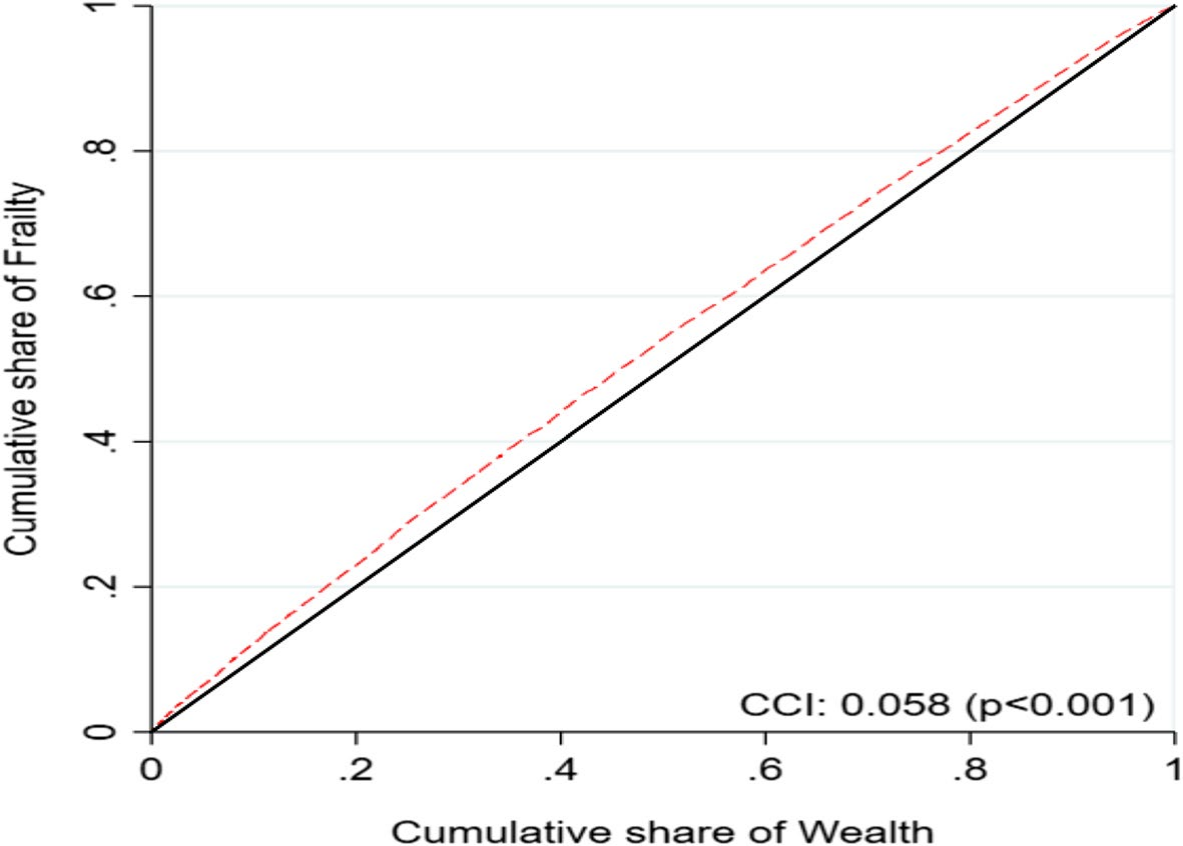
Concentration curve (CC) for wealth-related inequalities in frailty among Indian older adults, 2017–18

Table 4 presents the unadjusted and adjusted logistic regression estimates for frailty among Indian older adults. In model-I, older adults in the poorest group were 41% more likely to be frail than those in the richest category [OR = 1.41; 95% CI: 1.31, 1.52]. Similar results were obtained after controlling various individual, behavioral, health, and household factors in model-II [AOR = 1.23; 95% CI: 1.11, 1.38]. Figure 1 shows the concentration curve (CC) for wealth-related inequalities in frailty among older adults. The CC curve lies above the 45-degree line of equality, indicating that frailty was concentrated amongst older adults in the poor group [CI: 0.058].

**Table 4:** Logistic regression estimates for frail older adults by their background characteristics in India, 2017–18

Background characteristics	Model-1	Model-2
	UOR (95% CI)	AOR (95% CI)
**Wealth Index**		
Poorest	1.41*(1.31,1.52)	1.23*(1.11,1.38)
Poorer	1.27*(1.18,1.38)	1.19*(1.07,1.32)
Middle	1.17*(1.08,1.27)	1.09 (0.98,1.20)
Richer	1.13*(1.04,1.22)	1.03 (0.94,1.12)
Richest	Ref.	Ref.

Model-2 represents the Adjusted odds ratio (AOR). The model was adjusted for Individual, Behavioral, Health and Household factors; *CI* Confidence interval; * if *p* < 0.05

Table 5 presents the decomposition analysis results and shows the contribution of various individual, behavioral, health, and household predictors of the respondents in explaining socio-economic inequality in frailty among Indian older adults. The first two columns show the elasticities and concentration index (CI) for each predictor. The rest of the columns show each predictor’s absolute contributions and total percentage contributions to economic inequalities in frailty. The value of the absolute contribution depicts the extent of inequality contributed by a particular explanatory variable. Findings from Table 5 suggest that body mass index, wealth index, educational status, and region were the major and significant contributors to the socio-economic status (SES) related inequalities in frailty, followed by self-rated health and living arrangements. Nearly 45.2% of socio-economic status-related inequality in frailty was contributed by body mass index. Respondent’s educational status was responsible for around 18.1% of inequality in frailty. Moreover, the wealth index and region of residence contributed to about 28.4 and 14.4% of the socio-economic (SES) related inequality, respectively.

**Table 5:** Decomposition estimates of physical frailty among older adults by their background characteristics in India, 2017–18

Background characteristics	Elasticity	CCI	Absolute contribution	Percentage contribution	
**Individual factors**					
**Age**					
Young-old (60–69 years)					0.3
Old-old (70–79 years)	0.029	−0.006	0.000	0.9	
Oldest-old (80+ years)	0.023	0.005	0.000	−0.5	
**Sex**					
Male					−0.5
Female	−0.010	− 0.011	0.000	− 0.5	
**Education**					
No education/primary not completed					18.1
Primary completed	0.002	0.161	0.000	−1.3	
Secondary completed	−0.006	0.369	− 0.002	11.0	
Higher and above	−0.003	0.601	−0.002	8.4	
**Living arrangements**					
Alone					9.8
With spouse only	−0.008	−0.166	0.001	−6.4	
With children only	−0.035	0.098	−0.003	16.0	
Others	0.001	−0.091	0.000	0.2	
**Marital status**					
Currently married					0.7
Widowed	0.009	−0.023	0.000	0.9	
Others	−0.001	−0.072	0.000	−0.3	
**Working status**					
Never worked					−19.0
Currently working	−0.037	−0.165	0.006	−28.5	
Currently not working	0.023	−0.070	−0.002	7.4	
Retired	−0.001	0.458	0.000	2.1	
**Social engagement**					
No					4.8
Yes	−0.040	0.026	−0.001	4.8	
**Behavioral factors**					
**Tobacco consumption**					
No					−5.2
Yes	−0.009	−0.130	0.001	−5.2	
**Alcohol consumption**					
No					0.0
Yes	0.000	−0.081	0.000	0.0	
**Health factors**					
**Body mass index**					
Underweight					45.2
Normal	−0.030	−0.008	0.000	−1.1	
Overweight/obese	−0.021	0.334	−0.007	46.3	
**Self-rated health**					
Good					10.3
Poor	0.036	−0.062	−0.002	10.3	
**Difficulty in ADL**					
No					5.3
Yes	0.023	−0.051	−0.001	5.3	
**Difficulty in IADL**					
No					5.7
Yes	0.023	−0.052	−0.001	5.7	
**Morbidity status**					
0					−10.5
1	0.005	0.039	0.000	−0.8	
2+	0.010	0.209	0.002	−9.7	
**Household factors**					
**Wealth Index**					
Poorest					28.4
Poorer	−0.003	−0.307	0.001	−4.7	
Middle	−0.003	0.058	0.000	0.9	
Richer	−0.004	0.415	−0.002	8.3	
Richest	−0.007	0.770	−0.005	23.9	
**Religion**					
Hindu					0.5
Muslim	0.003	0.012	0.000	−0.2	
Christian	0.000	0.010	0.000	0.0	
Others	−0.001	0.212	0.000	0.7	
**Caste**					
Scheduled Caste					2.6
Scheduled Tribe	0.000	−0.351	0.000	−0.7	
Other Backward Class	−0.005	0.019	0.000	0.4	
Others	−0.003	0.213	−0.001	2.9	
**Place of residence**					
Rural					−10.9
Urban	0.005	0.452	0.002	−10.9	
**Region**					
North					14.4
Central	0.008	−0.187	−0.002	7.1	
East	0.010	−0.215	− 0.002	10.4	
Northeast	−0.001	−0.013	0.000	0.0	
West	−0.004	0.097	0.000	2.0	
South	0.006	0.175	0.001	−5.0	
**Calculated CCI**			−0.022	100.0	
**Actual CCI**			−0.058		
**Residual**			−0.036		

*CCI* Concentration index

## Discussion

Our results showed substantial socioeconomic disparities in frailty risk among community-dwelling older adults in India. A cross-sectional link between lower SES and frailty has been documented in multiple studies [11, 23, 46–49]. Similarly, older adults belonging to the poorest category had higher risk of frailty than those from rich households; education, living arrangements, BMI status and health variables including SRH, multimorbidity, ADL/IADL difficulties explained most of this disparity.

Longitudinal studies have revealed that lower education predicted a higher risk of frailty in older adults, and in some studies, lower educated as compared with higher educated older adults had a higher risk of worsening in the state of frailty [10, 14, 50]. Consistent with the literature, we found a higher risk of measured frailty in lower educated older individuals. However, the contribution of educational gradient in frailty was slightly lesser than the household economic gradient. On the other hand, the observed contribution of education to the socioeconomic inequalities in late-life frailty in our study can be explained by the fact that education can have positive impact on the attitudes and health behaviors which in turn can influence the decisions regarding the health of the older people [48]. Similarly, economically poor individuals becoming more frail compared to those from higher economic background may be attributed to the financial limitations that can limit the use of health care resources whereas, having adequate resources may help older people with early detection of age-related physical limitations and compensate for their incapacities by spending on specific health services that can be availed at home and other care facilities [51]. Nevertheless, the possibility of a reverse causation, where health status may determine the socioeconomic position of the people also should be taken into account while interpreting the findings.

Social engagements appear to reduce the rate of physical deficits in older populations, as has been observed in previous studies [52, 53], and social frailty is known to be a major risk factor for physical frailty [54]. In parallel with these findings, socially engaged older individuals in the present study had lower prevalence of frailty and the social engagement contributed to wealth-based inequality in frailty among older adults. According to the social capital theory, social engagement is an important tool for older adults to obtain the social resources which will have a positive impact on health and well-being among older people [55]. At the same time, marriage is considered as a central resource for social support for older individuals due to the narrowing of social networks in later life [56]. Supporting this, current results showed that the prevalence of frailty was higher in widowed older adults among poor and non-poor alike and the contribution of marital status to the observed inequality in frailty was substantially low.

In the earlier studies, there is evidence of the social gradient in the risk of tobacco use and heavy alcohol drinking which are found to be related to developing physical frailty [57, 58]. However, in our study, tobacco use and alcohol consumption accounted for very little of the frailty risk in old age which needs to be further investigated. Regarding our results on the contribution of BMI status towards the inequalities in frailty, they are in line with studies that reported that there is a strong association between BMI and physical frailty and the risk is higher among socioeconomically poor older adults [59, 60]. Although it was studied with the cross-sectional design which prohibits any causal inferences, the current finding suggests that maintaining a healthy body weight throughout individuals’ life course is important for healthy aging. With regards to other health factors, results followed the same direction although SRH explained 10.3% of the excess risk of frailty linked to a poor rating of health. Similarly, functional difficulties in ADL and IADL together contributed to 11% of the observed inequality in physical frailty in old age, suggesting that a broader assessment of frailty by considering deficits in multiple health and functional domains may bring out different results. Furthermore, possible pathways explaining the deleterious effects of socioeconomic adversity on physical frailty including cultural and environmental factors have to be further investigated.

The major strength of this study is the sizeable sample of older population which is nationally-representative. The large number of potential confounders considered in the study adds to the merits of the study. However, this study considered only cross-sectional relationships between socioeconomic and health characteristics of the participants and their physical frailty. Temporal relationships between the onset of frailty as an outcome and socioeconomic characteristics and the absolute contributions of several factors to the poor and non-poor disparities in frailty were not examined in the present study. Thus, further studies with follow-up data from upcoming waves of LASI survey are required. Moreover, although the association between socioeconomic status and weight change among people in developing countries is inconsistent across studies [61], the item of weight loss in the physical frailty may capture some information about the household economic status (i.e. the respondent did not have enough food in the household), which could be driving some of the associations between the primary exposure and the outcome in this study. Future research should also consider psychosocial variables such as mistreatment, everyday discrimination, social networks and cognitive functioning, and further anthropometric biomarkers including waist circumference and waist-hip ratio among other factors.

## Conclusion

The study found a higher risk of physical frailty in older adults with poor socioeconomic status. This disparity was predominantly explained by lower levels of education, adverse BMI status, poor SRH, difficulty in ADL and IADL and higher rates of multimorbidity in older individuals with lower socioeconomic status. Results suggest the need for formulating effective prevention and intervention strategies to decelerate the development of physical frailty among older adults in India, especially those with poor socioeconomic background.

## Supplementary Information

**Additional file 1.**

## Data Availability

The study uses secondary data which is available on reasonable request through https://www.iipsindia.ac.in/content/lasi-wave-i.

## Abbreviations

AOR: Adjusted odds ratio
CI: Confidence interval
CCI: Concentration index
BMI: Body mass index
ADL: Activities of daily living
IADL: Instrumental activities of daily living
MPCE: Monthly per capita consumption expenditure
