# Supplementary material for: Wealth disparity and frailty among community-dwelling older adults in India
Source: BMC Public Health. 2022 Nov 18;22:2123. doi: 10.1186/s12889-022-14434-9 (PMC9675126; doi:10.1186/s12889-022-14434-9)
Supplement: Supplementary file 1 — Additional file 1. [file 12889_2022_14434_MOESM1_ESM.docx]

**Supplementary Material**

**Calculation of the wealth index in LASI**

The wealth index was calculated using variables related to household assets, amenities, and housing quality. For constructing the wealth index in the Longitudinal Ageing Study in India (LASI), we have utilized a similar approach that is being used in Demographic Health Surveys (DHS). To construct a wealth index, we have utilized a set of 46 variables that cover the broad domains of the household's wealth and amenities and access to financial institutions. We have used principal component analysis (PCA) to construct the composite wealth index. We observed that the first principal component with an Eigenvalue of 7.2 has explained around 16 percent of the variance. The factor scores of the variables were used as the weight in constructing the overall composite index. The five wealth quintiles were derived from the overall composite score; poorest, poorer, middle, richer, and richest. These quintiles were based on the household's distribution, and population weight is adjusted for the household size in generating the composite wealth index. The reliability of the estimates has been carried out by alpha-test. The alpha value of 0.82 indicates the reliability of the wealth index. The description of the variables included in calculating the wealth index are as follows:

1. Housing Quality: number of rooms, separate kitchen, the monetary value of the house.
2. Household amenity: water and toilet facility in the household, availability of electricity, and cooking fuel.
3. Consumer durables: cars, scooters, motorcycles, bicycles, mopeds, refrigerators, computers, washing machines, laptops, stereo systems, cameras, camcorders, fans, coolers, air conditioners, mobile phones, musical instruments, jewelry, precious metals (gold, silver) and ornaments, antiques, valuable paintings, televisions, sewing machines, radios/transistors, water purifiers, juicer & mixtures, microwave oven.
4. Others: saving accounts, postal accounts, certificates of deposits or other depository products, stocks, mutual funds or shares in companies, and bonds.

The wealth quintile was categorized as *poor* which includes the poorest and poorer category and *non-poor* which includes the middle, richer and richest.

**Logistic regression: mathematical expressions**

In mathematical expression, the basic form of the logistic regression model describes the probability of the occurring of an event which is given as:

$$p=\frac{1}{1+e^{-Z}}= \frac{e^{Z}}{1+e^{Z}}----(1)$$

Where Z is the linear combination of the predictors and e is the base of the natural logarithm, equals 2.71828. The linear function Z can be written as:

$$Z=b_{0}+b_{1}x_{1}+b_{2}x_{2}\ldots\ldots\ldots+b_{k}x_{k}----(2)$$

By using equations (1) and (2), the function of the logistic regression is given as:

$$p=\frac{1}{1+e^{-(b_{0}+b_{1}x_{1}+b_{2}x_{2}\ldots\ldots\ldots+b_{k}x_{k})}}$$

On the other hand, the probability of non-occurrence of the event is given as:

$$1-p=1-\frac{1}{1+e^{-Z}}= \frac{e^{-Z}}{1+e^{-Z}}-----(3)$$

From equations (2) and (3),

$$\frac{1}{1-p}=e^{Z}-----(4)$$

The quantity $(\frac{1}{1-p})$ is called the odds of the happening of an event, generally denoted as Ω (uppercase omega).

$$Odds=\frac{1}{1-p}=Ω$$

The quantity $log(\frac{1}{1-p})$ is called the *log odds* or the log odds of *p*. Thus,

$$logit p=log\frac{1}{1-p}=logΩ$$

With the abovementioned expressions, the linear form of the logit function is given as:

$$logit p=b_{0}+b_{1}x_{1}+b_{2}x_{2}\ldots\ldots\ldots+b_{k}x_{k}$$

$$log\frac{p}{1-p}=b_{0}+b_{1}x_{1}+b_{2}x_{2}\ldots\ldots\ldots+b_{k}x_{k}$$

In the logit model, the coefficient ‘b’ is the value of change in the odds with one unit increase in the predictor. If the value of ‘b’ is positive, then the odds ratio tends to increase, and this factor will be greater than 1. On the other hand, if the value of ‘b’ is negative, then the odds ratio decreases, and its value is less than 1 but greater than zero. When ‘b’ is 0, the factor exponential of ‘b’ equals 1; therefore, the odds ratio remains unchanged.
